# Hybridization and embryological patterns underpinning reproductive barriers in *Dactylorhiza* (Orchidaceae)

**DOI:** 10.1038/s41598-025-24871-2

**Published:** 2025-10-22

**Authors:** Aleksandra M. Naczk, Małgorzata Kapusta, Bożena Kolano, Joanna Znaniecka, Katarzyna Meyza, Marcin Górniak, Joanna Rojek

**Affiliations:** 1 Department of Plant Taxonomy and Nature Conservation, Faculty of Biology, University of Gdańsk, Wita Stwosza 59, 80 308 Gdańsk, Poland; 2 Bioimaging Laboratory, Faculty of Biology, University of Gdańsk, Wita Stwosza 59, 80 308 Gdańsk, Poland; 3 Plant Cytogenetics and Molecular Biology Group, Institute of Biology, Biotechnology and Environmental Protection, Faculty of Natural Sciences, University of Silesia in Katowice, Katowice, Poland; 4 Department of Plant Protection and Biotechnology, Intercollegiate Faculty of Biotechnology, University of Gdańsk and Medical University of Gdańsk, Abrahama 58, 80 307 Gdańsk, Poland; 5 Department of Genetics, Faculty of Biological Sciences, Kazimierz Wielki University, J.K. Chodkiewicza 30, 85 064 Bydgoszcz, Poland; 6 Department of Evolutionary Genetics and Biosystematics, Faculty of Biology, University of Gdańsk, Wita Stwosza 59, 80 308 Gdańsk, Poland; 7 Department of Plant Experimental Biology and Biotechnology, Faculty of Biology, University of Gdańsk, Wita Stwosza 59, 80 308 Gdańsk, Poland

**Keywords:** *Dactylorhiza*, FISH, Hybridization, Ovule development, Reproductive isolation, Zygotic barriers, Developmental biology, Genetics, Plant sciences

## Abstract

Pre- and postmating barriers are strongly associated with embryological processes occurring in developing flower buds and flowers. Although the embryology of *Dactylorhiza* representatives has been briefly described, comprehensive cytoembryological studies are scarce. In this study, the mechanisms of reproductive isolation within the *Dactylorhiza incarnata/maculata* complex were examined using cytological, embryological, and molecular approaches. Four taxa were analyzed: the diploid ancestors *D. incarnata* and *D. fuchsii*, the autotetraploid *D. maculata*, and the allotetraploid *D. majalis*. Fluorescent in situ hybridization revealed species-specific rDNA patterns, with *D. majalis* exhibiting rDNA patterns consistent with the hypothesis of its allopolyploid origin. Embryological assessments under controlled pollination conditions demonstrated variable gametophyte development and seed viability, with diploids and *D. maculata* showing 73–85% seed viability compared to 28% for *D. majalis.* Interspecific crosses between diploid progenitors yielded asymmetrical hybrids, with crosses between *D. fuchsii* × *D. incarnata* showing a significantly higher success rate. Cytological analysis revealed meiotic abnormalities, including chromosome segregation disruptions during megasporogenesis in *D. incarnata* and *D. maculata*, suggesting that cytogenetic mechanisms may reduce seed fertility and viability. Analysis of microsatellite markers of F_1_ hybrids revealed maternal influence on hybrid genotypes. All these findings suggest that postzygotic barriers dominate over prezygotic mechanisms in this complex, challenging previous assumptions about the direction of allopolyploids formation within the group. Our results shed new light on the reproductive mechanisms underlying the recurrent hybridization and polyploidization events that have shaped diversity in the *Dactylorhiza* genus.

**Supplementary Information:**

The online version contains supplementary material available at 10.1038/s41598-025-24871-2.

## Introduction

Most flowering plants rely on sexual reproduction through seeds. Female and male gametophytes produce gametes for fertilization and subsequent formation of the embryo and endosperm (seed). Deregulation of these processes can lead to seed loss, but new strategies for regaining population fertility may also emerge. Among many factors, hybridization has been described as a major trigger for various reproductive changes [e.g.,^1,2^].

Reproductive barriers, and thus isolation, are essential for maintenance of species boundaries [e.g.,^3–6^]. The integrity of a species, particularly among closely related species, depends on the strength of individual reproductive isolation. In flowering plants, these barriers can function before or after pollination and are therefore typically classified as pre- and postmating barriers. Premating barriers encompass geographic, temporal, and pollinator isolation, while postmating barriers may result from prezygotic (pollen–stigma and sperm cells–ovule interactions) and postzygotic (embryo mortality, germination failure, and lack of viability and sterility of hybrids and seeds) mechanisms^7,8^.

Approximately one-third of orchid species are deceptive and do not offer any rewards to their pollinators^9–11^. Food deception is a pollination strategy in which flowers lure pollinators by imitating reward signals without offering the reward. This mechanism frequently attracts diverse, rather non-specific pollinators, and facilitates the occurrence of hybridization^9,12–14^. Comparative analyses have shown that prezygotic isolation barriers in food-deceptive orchids are generally weak^3,15,16^. Postzygotic barriers appear to be quite substantial, although in many cases insufficient to prevent hybridization. The extent to which natural hybridization occurs is determined by the relative strength of these various reproductive barriers and differs greatly between species^17^.

In the case of the *Dactylorhiza* genus, its deceptive pollination system is well documented, and are characterized by weak premating isolation, but show strong evidence for intrinsic postmating reproductive barriers, particularly for late-acting postzygotic barriers [e.g.,^3,15,16,18–21^]. In addition, recurrent hybridization and polyploidization have been identified as factors leading to high diversity within the *Dactylorhiza* genus^22–27^. Species belonging to this genus are mostly diploid or tetraploid, their distribution range extends from Europe to East Asia, and most of them belong to the *Dactylorhiza incarnata/maculata* polyploid complex. Most taxa within this complex are classified as belonging to the *D. majalis/traunsteineri* allotetraploid group, which arose as a result of multiple and independent hybridizations between the diploid *D. incarnata* s.l. (considered the paternal lineage/pollen parent) and *D. maculata* s.l. (recognized as the maternal lineage/ovule parent), with the diploid *D. maculata* subsp. *fuchsii* and the autotetraploid *D. maculata* subsp. *maculata*^24,25,28–35^. However, the processes accompanying the formation of new tetraploids within this complex are not yet fully understood, as are the underlying embryological and cytological mechanisms. As noted by De hert et al.^21,36^, reproductive isolation between *Dactylorhiza* species may be asymmetrical, depending on which *Dactylorhiza* species acts as the ovule- or pollen-providing parent. Similar patterns of asymmetry have also been reported in other plants^3,37–39^. These findings challenge the widely accepted model of allopolyploid origin of many tetraploid *Dactylorhiza* species, which assumes that *D. incarnata* consistently acted as the pollen parent rather than the ovule parent^31^. This suggests that the mechanisms underlying the formation of these taxa may be more complex and diverse than previously thought.

Both pre- and postmating barriers are strongly linked to embryological processes occurring in developing flower buds and subsequently in flowers. Although the embryology of *Dactylorhiza* representatives has been briefly described^40–44^, detailed cyto-embryological studies are still lacking.

In the present study, cytological and embryological studies were applied to characterize the flowers’ development before and after pollination in four representatives of the *Dactylorhiza incarnata/maculata* complex and their hybrids. Crossing experiments with additional microsatellite markers analysis were carried out with the following objectives: (1) to estimate the strength and nature of reproductive barriers; (2) to characterize the role of postmating barriers between the studied taxa; and (3) to describe the degree of in situ hybridization among the four *Dactylorhiza* species, and in particular between *D. incarnata* × *D. fuchsii* in both directions*.*

## Materials and methods

### Plant material

For this study, we selected four *Dactylorhiza* taxa within the *Dactylorhiza incarnata/maculata* complex, which represented diploid progenitors and their tetraploid derivatives: the parental lineages—*D. incarnata* var. *incarnata* (L.) Soó (2*n* = 2*x* = 40) and *D. maculata* subsp. *fuchsii* (2*n* = 2*x* = 40); autotetraploid with four homologous chromosome sets—*D. maculata* subsp. *maculata* (L.) Soó (2*n* = 4*x* = 80); and fixed allotetraploid between *D. incarnata* × *D. maculata* subsp. *fuchsii*—*D. majalis* subsp. *majalis* (Rchb.f.) P.F. Hunt & Summerh. (2*n* = 4*x* = 80)^32,45^. The presented taxonomic classification within the *Dactylorhiza incarnata/maculata* complex is in accordance with Hedrén’s concept^29^. However, for clarity, we will use the shorter notation of these taxa, i.e., *D. incarnata*, *D. fuchsii*, *D. maculata*, and *D. majalis*, without taking into account the taxonomic complexities associated with the *Dactylorhiza incarnata/maculata* complex. The most common number of chromosomes is given in brackets after the species name.

Plants grew in four monospecific populations in Gdańsk Pomerania, northern Poland: (1) *D. incarnata*, Mechowiska Sulęczyńskie—54° 13′ 56″ N 17° 46′ 27″ E, Naczk/2015/R1-R9/UGDA; (2) *D. majalis*, Mechelińskie Łąki—54° 36′ 32″ N 18° 30′ 44″ E, Naczk/2015/R10-R15/UGDA; (3) *D. fuchsii*, Łęg nad Swelinią—54° 27′ 41″ N 18° 32′ 07″ E, Naczk/2015/R16-R20/UGDA; (4) *D. maculata*, Piaśnickie Łąki—54° 49′ 25″ N 18° 03′ 38″ E, Naczk/2015/R21-R25/UGDA. Aleksandra Naczk undertook the formal identification of the plant material used in the study. Reference specimens for each sample were deposited at the Herbarium of the University of Gdańsk, Faculty of Biology, University of Gdańsk (UGDA, Poland).

### Chromosome counting

For cytogenetic analysis, root tips were pretreated with 2 mM 8-hydroxyquinoline for 4 h (h) at room temperature and then fixed in 3:1 ethanol/acetic acid. Fixed roots were washed in a 0.01 M citrate buffer (pH 4.8) and subjected to enzymatic maceration in a mixture of 20% pectinase (Sigma P0690) and 4% cellulose (Onozuka R-10 Serva) for 2.5–4 h at 37 °C. A single root tip was rinsed with cold distilled water and placed into a drop of 45% acetic acid on a microscope slide and then squashed. The coverslips were removed after freezing and the slides were air-dried. The number of chromosomes was determined for three individuals from each taxon. Karyotype analyses were performed on well-spread metaphase plates stained with 2 μg/mL DAPI (4ʹ,6-diamidino-2-phenylindole) (Sigma) in Vectashield mounting medium (Vector Laboratories, Peterborough, UK), using at least five plates for each species.

Fluorescent in situ hybridization (FISH) was used to analyse the number and chromosomal localisation of rRNA gene loci in the *Dactylorhiza incarnata/maculata* complex. FISH was performed according to the protocol described by Yücel et al.^46^. A 2.3-kb fragment of the 25S rDNA coding region of *Arabidopsis thaliana*^47^, labelled with digoxigenin-11-dUTP (Roche, Basel, Switzerland), was used to detect 35S rDNA loci. The 5S rDNA monomer, isolated from *Triticum aestivum*^48^ and labelled with tetramethyl-rhodamine-5-dUTP (Roche, Basel, Switzerland), was used to detect 5S rDNA loci. Both probes were labelled using nick translation, following the manufacturer’s instructions (Roche, Basel, Switzerland). The hybridization mixture consisted of 100 ng of each DNA probe, 50% formamide, 2 × SSC, and 10% dextran sulfate. Chromosome spreads and the hybridization mixture were denatured together at 72 °C for 5 min and incubated for 48 h in a humid chamber at 37 °C. Stringent washes were performed in 0.1 × SSC at 42 °C for 10 min (stringency 70%), followed by detection of digoxigenin using a fluorescein-5-isothiocyanate (FITC)-conjugated primary antibody (Roche) and a FITC-conjugated anti-sheep secondary antibody (Jackson ImmunoResearch, Pennsylvania, USA). Chromosome slides were counterstained with 2 μg/mL DAPI (Sigma) in Vectashield mounting medium (Vector Laboratories, Peterborough, UK) and analyzed using an AxioImager.Z2 fluorescence microscope equipped with an AxioCam HRm monochromatic camera (Zeiss, Germany).

### Embryological survey of flowers under open pollination conditions (‘open’)

In spring 2015, four plants in the young flower bud stage (one plant of each studied species) were placed in an experimental plot at the University of Gdańsk to check fruit set. The remaining plants (5 plants per species) were displaced to controlled conditions in the greenhouse at the University of Gdańsk (Poland). Flower buds and young flowers, immediately after opening (Supplementary Fig. S1A–D), were marked and analyzed 0, 24, 48 h after anthesis (HAA), and after 5 and 10 days (DAA); the mature seed bags were collected from the experimental plot. After fixation in Clarke’s solution, standard paraffin and clearing methods, and callose detection were applied according to the protocols of Rojek et al.^49,50^. A total of 12 flower buds (3 flower buds per species) and 80 flowers were analyzed (20 flowers × 4 species). An average of 500 ovules per ovary were analyzed.

### Parthenogenesis experiment

To verify the occurrence of any apomeiotic and/or parthenogenetic events, the flowers of *D. incarnata* were emasculated immediately after anthesis. The flowers were then isolated from foreign pollination (mesh bag; Supplementary Fig. S1E) and left unpollinated for 48 h after emasculation (HAE), 5 days after emasculation (DAE) and 10 DAE. The flowers were then collected and subjected to the same analysis as the open-pollinated flowers. Five flowers and an average of 500 ovules per ovary were analyzed.

### Hand-pollination experimental crosses

To assess the strength of postzygotic reproductive barriers, a series of hand-pollination experiments were conducted (Supplementary Fig. S1F–H). The study focused exclusively on three species belonging to the *Dactylorhiza incarnata/maculata* complex (*D. incarnata*, *D. fuchsii* and *D. majalis*). This choice is attributable to the results of numerous molecular studies, which showed that the allotetraploid *Dactylorhiza* species originated from hybridization of *D. incarnata*, considered the paternal lineage and *D. fuchsii*, recognized as the maternal lineage [e.g.,^25,28,31^). Consequently, in order to assess the potential functionality of both diploid parental species as ovule and/or pollen parents, an additional cross was carried out, specifically a cross between *D. incarnata* ♀ × *D. fuchsii* ♂.

Experimental crosses under similar conditions were carried out in the Greenhouse of Gdańsk University (Poland) in spring 2015. Crosses were performed in both directions, ensuring that each plant provided and received pollen. Six flowers were used for each pollination treatment and direction. Careful examinations were conducted to determine the presence of ovary swelling and fruit set in all treated flowers.

The following pollination treatments were carried out: (1) induced self-pollination (pollination with pollen from the same flower) = induced autogamy (only for *D. incarnata*); (2) cross-pollination with pollen from another individual of the same species = induced allogamy; (3) interspecific cross-pollination between diploids, as well as between diploids × allotetraploid taxa, in both directions.

Flowers were collected 24 and 48 HAP and 5, 10, and 21 DAP, as well as at the mature seed-bag stage. In the case of allogamy and interspecific cross-pollination, an additional control point (7 DAP) was used. They were then fixed and treated in the same way as for open pollination (see previous sections) or fixed in PFA/GA fixative solution (4%/0.25%) in PBS buffer and stored in 96% ethanol in the fridge until use. The preparation of flower buds and flowers was carried out in accordance with Steedman’s wax, as described by Krawczyk et al.^49^. The nuclear chromatin was then stained with 7 µg ml^−1^ DAPI (Sigma), and the cell walls were stained with 0.01% Calcofluor White and viewed using a Nikon Eclipse E800 and Leica DM 6000B.

### Seed viability

To estimate the viability of seeds from open pollination and induced allogamy, the tetrazolium method was employed [according to the protocols^50,51^], in which only viable embryos were stained red after the treatment. The percentage of viable seeds was estimated as the ratio of the number of stained seeds to the total number of seeds. Embryos exhibiting reddish colouration were classified as viable (fertile seeds), while those that were uncoloured or exhibited yellowish tissue colouration were considered non-viable (sterile seeds). The proportion of viable, non-viable and empty seeds was determined for approximately 100 randomly selected seeds of each species after allogamy and open pollination experiments.

### In vitro germination test

Seeds from reciprocal interspecific crosses between *D. incarnata* × *D. fuchsii* were introduced into in vitro cultures. Sterilization and cultivation conditions were in accordance with the protocol developed by Znaniecka & Łojkowska^52^, with minor modifications. Germination was performed on a Malmgren Modified Terrestrial Orchid Medium^53^. Seeds were surface sterilized by immersion in a 5% calcium hypochlorite Ca(OCl)_2_ for various time intervals (from 1 to 24 h). This was done to ensure scarification if necessary. Approximately 300 seeds were sown on a single Petri dish. After sowing, the seeds were stratified at 4 °C for 3 months. Germination and protocorm development were evaluated after 75 days of incubation at 20 °C in the darkness. The developed protocorms with visible young leaves were then collected and subjected to genotyping. For further genetic analysis, 35 randomly selected seedlings were used for each interspecific cross between diploid parental species.

### Genetic analysis

All individuals involved in hand-pollination crosses were genotyped to confirm the taxonomic identity. Total DNA was extracted from the samples of leaves following the method commonly known as 2 × CTAB^54^. Variation within the nuclear genome was examined by six microsatellite loci (ms3, ms6, ms8, ms10, ms11 and ms13) developed by Nordström & Hedrén^55^, while the plastid genome was examined using ten size-variable markers, which were documented by Hedrén et al.^45^. Plastid and nuclear microsatellites were used with PCR protocols reported in Naczk et al.^33,56^. Additionally, six nuclear SSR loci were included (D2, D8, D16, D51, D52, D55), as reported by Balao et al.^57^. In this case, the multiplex PCR reaction was carried out in 10 μL volumes, containing: 0.125 μM primer for each D2, D16 and D52 locus, 0.15 μM primer for D8 and D51 locus, 0.1 μM primer for D55 locus, and template DNA (1 ng/μL) in 2 × Multiplex PCR Master Mix (Qiagen). One of the primers used for amplification was dye-labelled at the 5ʹ-end (Life Technologies), to detect the amplified fragments. The touchdown protocol was used for the PCR amplification: the initial denaturation round (95 °C for 15 min) was followed by 9 cycles of 94 °C for 30 s, annealing at 63 °C (with a 1 °C reduction per cycle) for 1 min 30 s, and extension at 72 °C for 1 min. The next 24 cycles utilized 94 °C for 30 s, an annealing temperature of 53 °C. The final cycle used 72 °C for 10 min. The PCR products from each reaction were mixed with appropriate size standards to enable exact size determination (GeneScan 600 LIZ, Life Technologies). All samples intended for length-variable fragment analysis were genotyped on an ABI 3130xl genetic analyzer (Life Technologies). Fragment size was determined using Genescan and Genotyper v. 3.7. Seedlings from in vitro cultures, obtained from seeds derived from interspecific crosses (*D. incarnata* × *D. fuchsii* in both directions), were genotyped using the same markers and PCR conditions as described above. In summary, a total of 12 nuclear SSR loci and 10 plastid size-variable markers were examined (Supplementary dataset).

All obtained hybrid seedlings were treated as a single sympatric group after artificial hybridization crosses, with *D. incarnata* and *D. fuchsii* as reference material. To visualize genetic patterns among in vitro hybrids and diploid parental species, a principal coordinate analysis (PCoA) was performed based on Gower distance using PAST 4.17c^58^, and then also on Bruvo distance in GENODIVE 3.0^59^. The advantage of the Bruvo method^60^ is that it is suitable regardless of the ploidy of individuals and unaffected by allele dosage uncertainty^61^. Bayesian clustering analysis was performed in the STRUCTURE 2.3.4^62–64^. This model minimizes deviations from Hardy–Weinberg and linkage equilibrium. Individuals were assigned to two sources of unquestionable membership in the species considered parental and to a presumed hybrid pool after hybridization. Calculations assumed no prior population information, with correlated allele frequencies and an admixture model, with 500,000 burn-in steps and 750,000 MCMC iterations. Ten replicates were executed for *K* = 1–2 (parental species contribution), and then for *K* = 1–4 (accounting for maternal origin of hybrids: IF vs. FI). We also tested STRUCTURE with Popflag = 1 (known ancestry as learning samples). The value of *K* that best fits our data was selected using the Δ*K* statistic^65^ implemented on the StructureSelector web platform^66^. The Haplotype Analysis 1.05^67^ was used to detect the frequency and number of recorded plastid haplotypes.

## Results

### Number of chromosomes

**Fig. 1:**
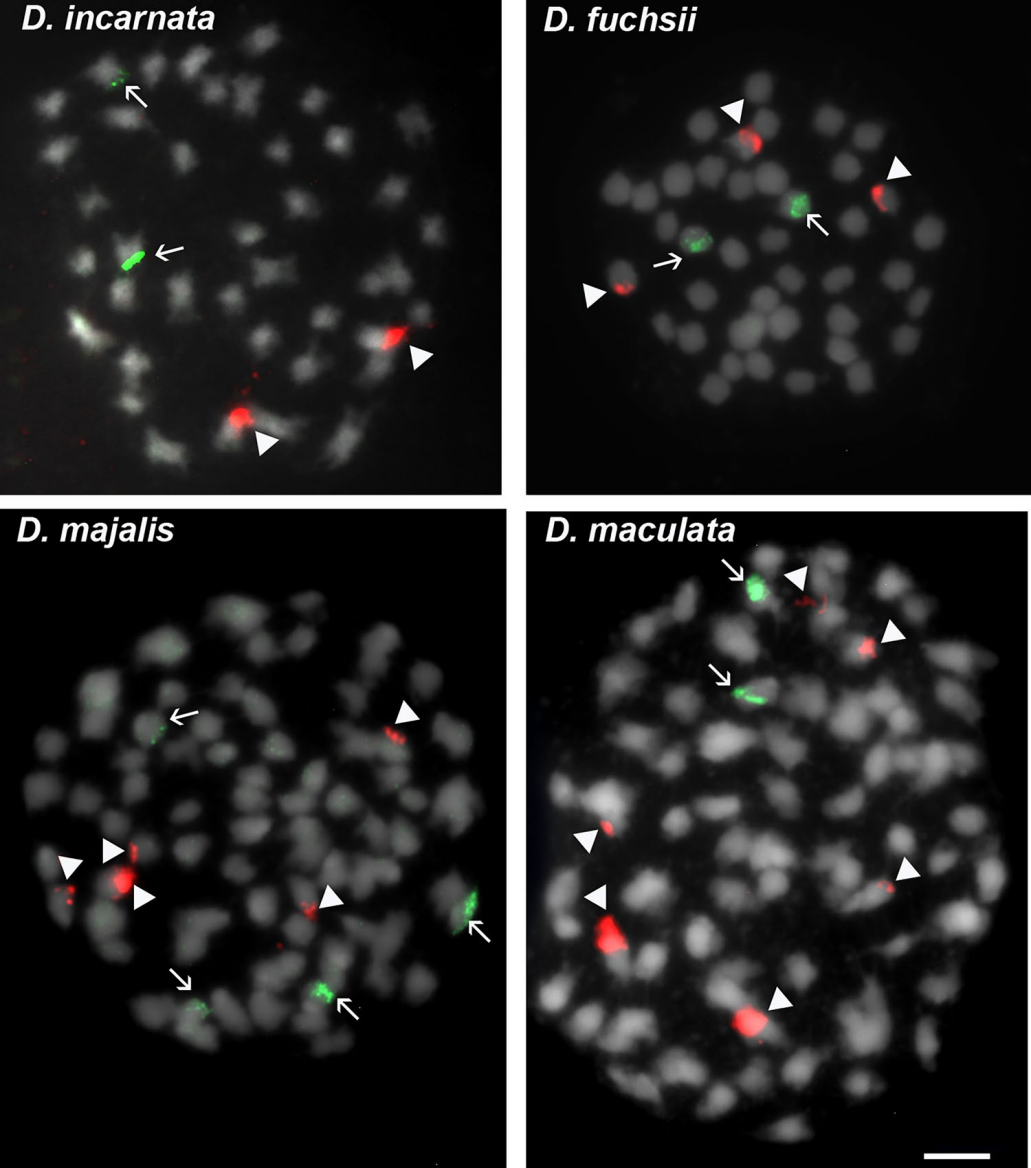
Distribution of the 35S and 5S rDNA loci on the mitotic metaphase chromosomes of the studied *Dactylorhiza* species. Fluorescence in situ hybridization was performed using 25S rDNA (red fluorescence) and 5S rDNA (green fluorescence) probes to examine the diploid species *D. incarnata* (paternal lineage) and *D. fuchsii* (maternal lineage), as well as the allotetraploid *D. majalis* and autotetraploid *D. maculata*. Arrows indicate 5S rDNA loci, while arrowheads indicate 35S rDNA loci. Scale bar: 5 µm.

Chromosome numbers were verified for the four analyzed species. As expected, *D. incarnata* and *D. fuchsii* exhibited 2*n* = 2*x* = 40 chromosomes, while *D. maculata* and *D. majalis* displayed 2*n* = 4*x* = 80 chromosomes (Fig. 1). The hypothetical ancestral species *D. incarnata* and *D. fuchsii* exhibited different patterns of hybridization signals after FISH with 5S rDNA and 25S rDNA as probes. In *D. fuchsii*, three 35S rDNA and two 5S rDNA signals were detected in subterminal positions on different chromosomes pairs. In the karyotype of *D. incarnata*, one locus each of 35S and 5S rDNA was observed (Fig. 1). Both loci were placed in subtelomeric positions on two different chromosome pairs. In the karyotype of autotetraploid species *D. maculata*, two major and four minor 35S rDNA signals were observed, all located in subtelomeric positions on the chromosomes. The locus of 5S rDNA was also observed in subtelomeric position on another pair of homologous chromosomes (Fig. 1). The second tetraploid species, *D. majalis*, showed five signals of 35S rDNA (one major and four minor) and two signals of 5S rDNA. All observed rDNA loci were placed in subterminal positions on different chromosomes (Fig. 1).

### Flower development under open pollination conditions

**Fig. 2:**
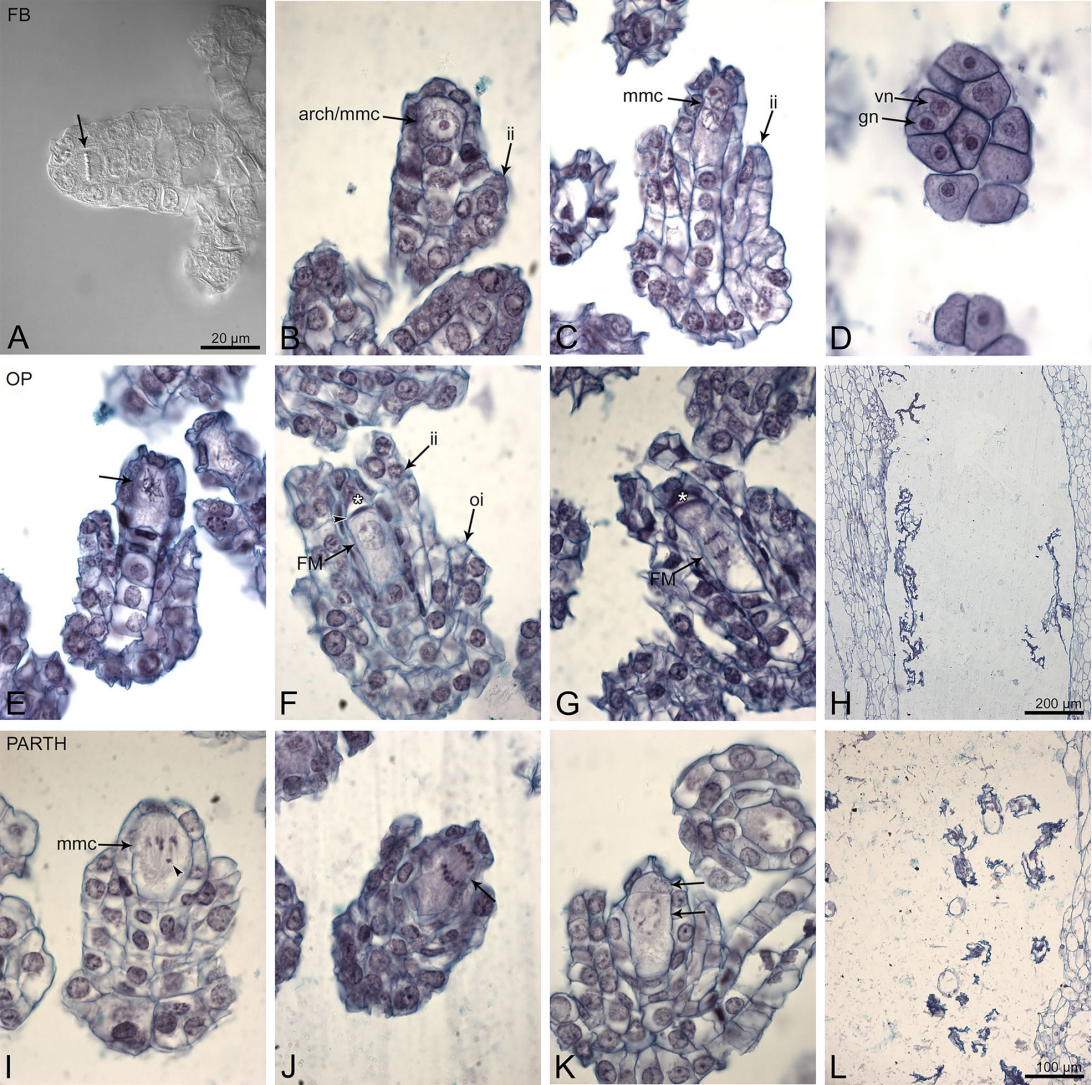
Embryological survey of the flower bud (**A**–**D**) and flowers from open pollination (**E**–**H**) and parthenogenesis (**I**–**L**) experiments for *D. incarnata*. In the case of open pollination, similar stages of embryo development were observed in the other species studied. (**A**) Division (arrow) in uppermost nucellar cell to create archespore. (**B**) The archesporial cell that started to differentiate into megaspore mother cell. (**C**) Mmc at prophase I of meiosis. (**D**) Two-cell pollen grain in pollinia. (**E**) Prophase I, 24 HAA. (**F**) End of megasporogenesis 48 HAA; the lower most positioned functional megaspore and the remains of megaspore triad/tetrad (star and arrowhead). (**G**) Start of megagametogenesis 5 DAA; the division in one-nucleate FM/FG; the remnants of degenerated megaspores are marked by star. (**H**) Empty ovary 10 DAA. (**I**) 24 HAE; irregular meiosis at metaphase I; losing bivalent/chromosome marked by arrowhead. (**J**) 48 HAE; anaphase I. (**K**) 48 HAE; the asymmetric dyad (arrow); the micropylar cell of the dyad is much smaller than chalazal one. (**L**) Degenerated ovule inside the ovary 10 DAE. arch-archesporial cell; mmc-megaspore mother cell; vn-vegetative nucleus; gn-generative nucleus; ii-inner integument; oi-outer integument; FM-functional megaspore; FG-female gametophyte. Bar = 20 µm for (**A**–**G**, **I**–**K**) images.

Firstly, we verified whether sexual reproduction of all four studied species proceeded without disruption, and then we conducted an embryological analysis. In the flower buds before anthesis or at anthesis stage, the ovules were at a very early stage of development (Fig. 2A–D). The internal integument was not developed or was in an early phase of differentiation. Most ovules contained nucellus with the archespore cell differentiated. Microsporogenesis occurs much earlier than megasporogenesis. In the bud, where only the female archespore was present, young pollen grains were already visible in the pollinium.

At 24–48 HAA, the ovules were at different stages of megasporogenesis: from megaspore mother cell (MMC) to functional megaspore (Fig. 2E–F). On day 5, the end of megasporogenesis was observed; triads were usually present that consisted of a functional chalazal megaspore (FM), a non-functional sister cell, and the remnants of the degenerated micropylar cell (Fig. 2G). Some ovules contained 1–2 cell embryo sacs. On day 10, most flowers remained unpollinated and contained degenerated ovules (Fig. 2H). Very few seed-bags collected from the experimental plot contained viable seeds (see *‘*Seed viability*’* section).

### Parthenogenesis and autogamy

Due to the limited flower accessibility and the latest reports of the impossibility of autogamy sensu stricto (spontaneous self-pollination), but with a high success rate of induced autogamy [manual self-pollination; see Wróblewska et al.^68^], we focused solely on the parental species *D. incarnata* to confirm previous findings.

#### Parthenogenesis

As in the open pollination experiment, at 24–48 HAE megasporogenesis was noted in the ovules (Fig. 2I–K). During megasporogenesis in *D. incarnata,* chromosomes/bivalent deletion from the metaphase I plate was observed (Fig. 2I). In the formed dyad, the chalazal cell of the dyad was generally larger; and the micropylar cell of the dyad degenerated (Fig. 2K). On day 5, the functional megaspore stage was dominant, with a few-nucleate embryo sacs being sporadically observed. On day 10, the ovary tissue was completely degenerated; gametophytes and embryos were absent (Fig. 2L).

#### Autogamy

**Fig. 3:**
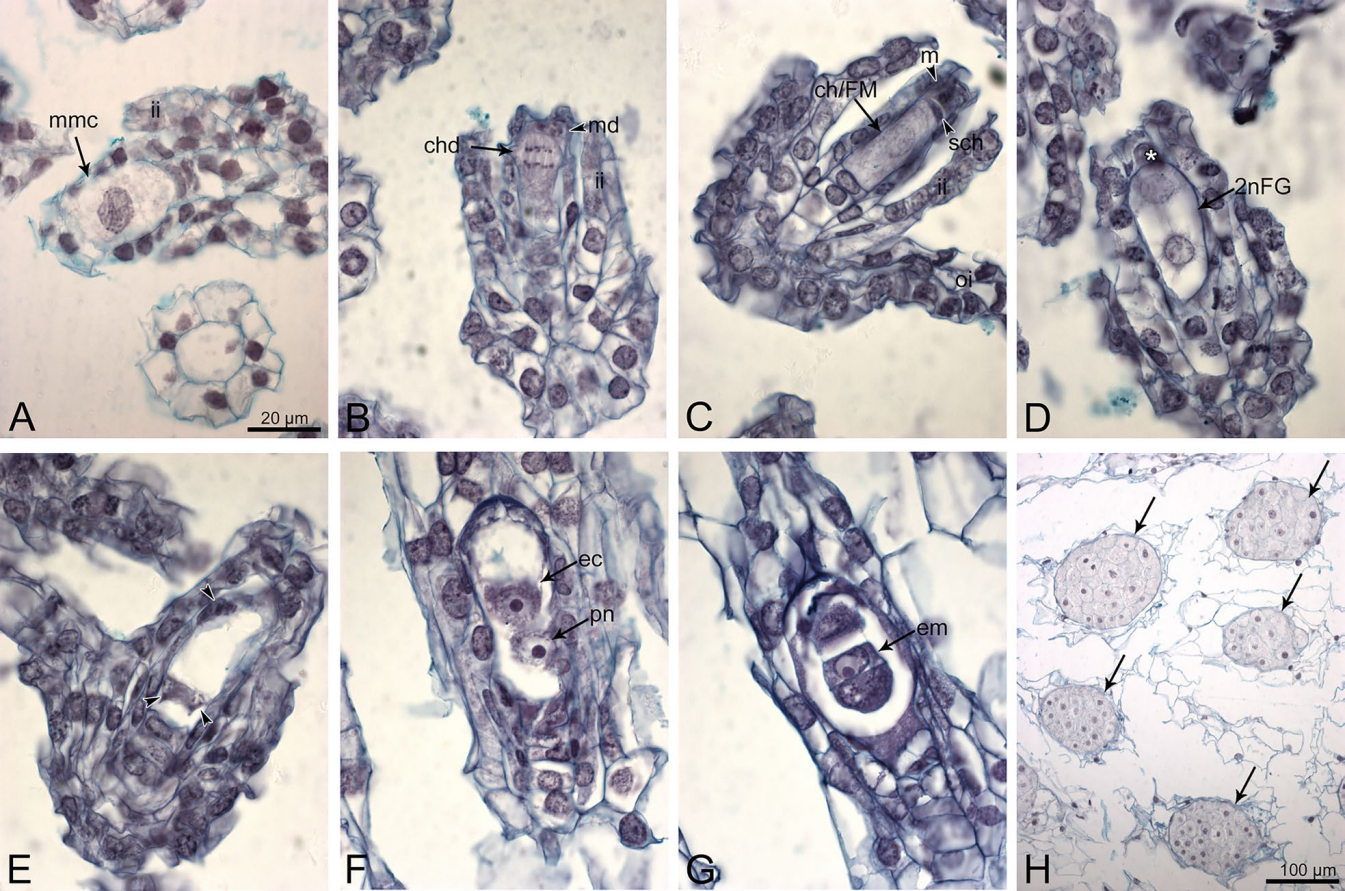
Autogamy in *D. incarnata.* (**A**) 24 HAP, megaspore mother cell at prophase I. (**B**) 24 HAP, division in the chalazal cell of the dyad (chd); the micropylar cell of the dyad (md) degenerates. (**C**) 48 HAP, end of megasporogenesis; triad of megaspores; this enlarged and chalazaly positioned becomes the functional megaspore (FM). (**D**) 48 HAP, early megagametogenesis: two nucleate FG; the remnants of degenerated megaspores are marked by a star. (**E**) 48 HAP, four-nucelate FG; three of four nuclei are marked by arrowheads. (**F**) 10 DAP; mature female gametophyte with the egg cell (ec) and the polar nucleus (pn). (**G**) 10 DAP, a few-cell embryo (em). (**H**) 21 DAP; globular embryos (arrows) inside ovary. mmc-megaspore mother cell; ii-inner integument; oi-outer integument; chd-chalazal cell of the dyad; md-micropylar cell of the dyad; ch-chalazal megaspore of the triad; FM-functional megaspore; sch-subchalazal megaspore of the triad; m-micropylar megaspore of the triad; FG-female gametophyte; ec-egg cell; pn-polar nucleus; em-embryo. Bar = 20 µm for (**A**–**G**) images.

At 24–48 HAP, the developmental desynchrony of the ovules was significant. Megasporogenesis stages dominated, but 2- or 4-nucleate female gametophytes were also observed (Fig. 3A–E). At 5 DAP, ovule and gametophyte development progressed. In 10-day-old ovaries, viable ovules contained either mature female gametophyte (Fig. 3F) or a zygote/few-cell embryo (Fig. 3G). On day 21 (21 DAP), the ovules mainly contained a globular embryo (Fig. 3H).

#### Allogamy

**Fig. 4:**
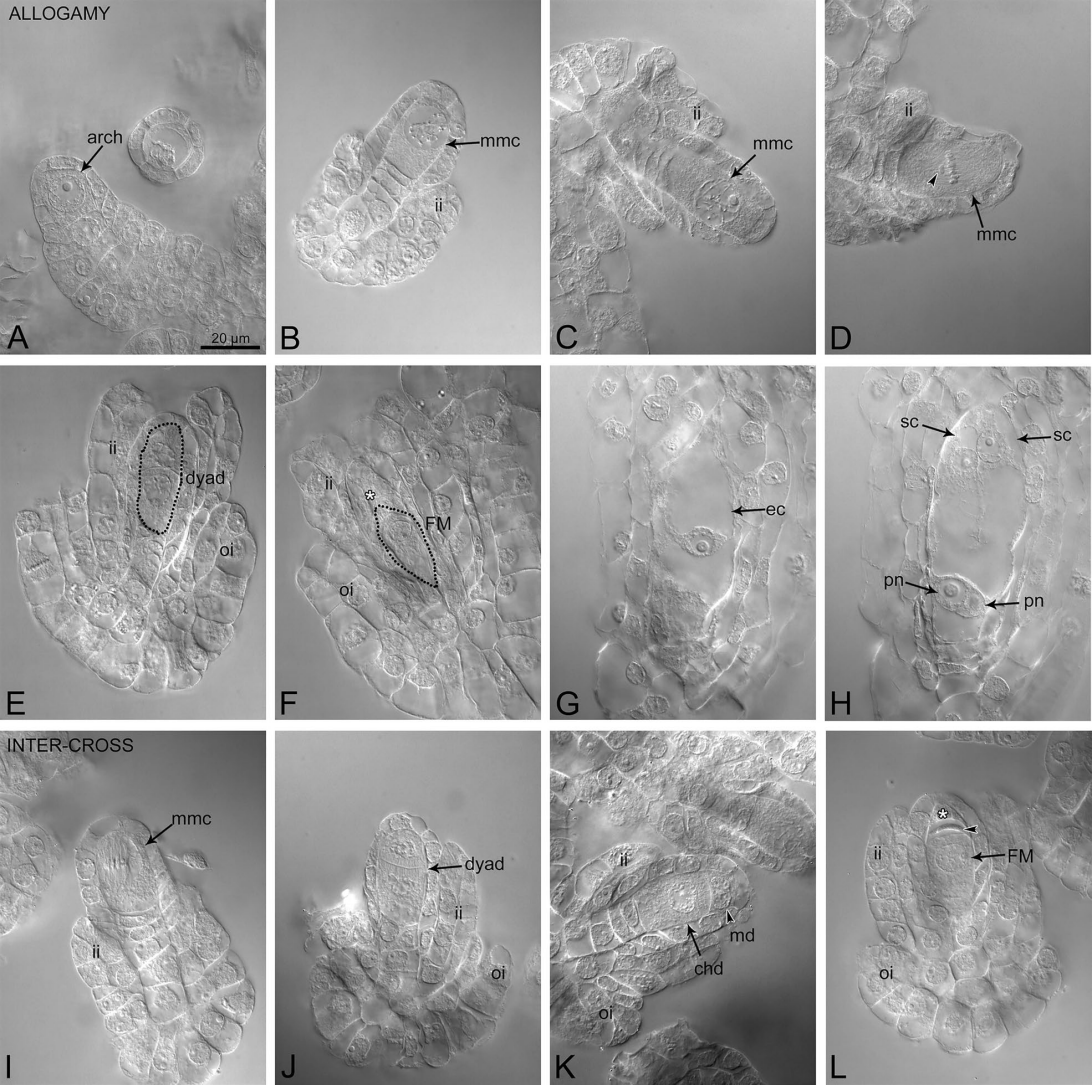
Representative photographs of the ovule development after allogamy and interspecific crossing. Allogamy (**A**–**H**); interspecific crossing (**I**–**L**). *D. fuchsii* (A,B,E,F); *D. majalis* (G,H); *D. maculata* (**C**, **D**). (**A**) 24 HAP, archespore cell. (**B**–**C**) 48 HAP, megaspore mother cell at prophase I. (**D**) 24 HAP, metaphase I, losing bivalent/chromosome marked by arrowhead. (**E**) 5 DAP, the dyad stage. (**F**) 5 DAP, the functional megaspore stage; the remnants of degenerated megaspores are marked by a star. (**G**–**H**) 5 DAP, mature FG with an egg cell (**G**), two synergid cells and two polar nuclei (**H**). (I) ♀ *D. fuchsii* × *D. incarnata* ♂ 24 HAP, metaphase I. (**J**–**K**) ♀ *D. incarnata* × *D. majalis* ♂ 24 HAP, the dyad stage. (**L**) ♀ *D. incarnata* × *D. majalis* ♂ 24 HAP, the functional megaspore stage. Similar ovule developmental stages were observed for all tested species. mmc-megaspore mother cell; ii-inner integument; oi-outer integument; chd-chalazal cell of the dyad; md-micropylar cell of the dyad; ; FM-functional megaspore; ec-egg cell; pn-polar nucleus. Bar = 20 µm for all images.

**Fig. 5:**
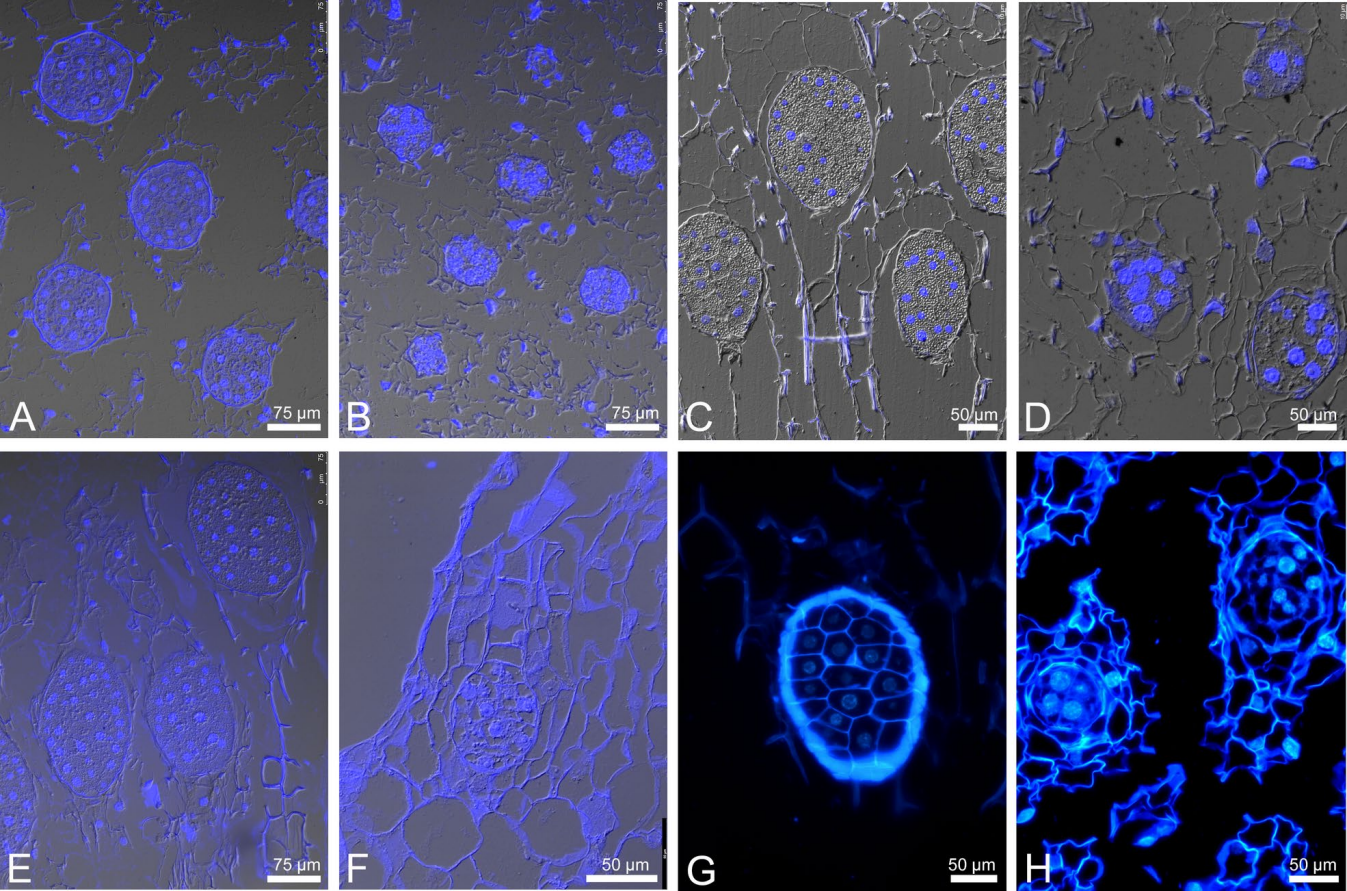
Embryos formed 21 DAP. (**A**–**D**) allogamy. (**E**–**H**) Interspecific crossing. (**A**) *D. incarnata*. (**B**) *D. fuchsii*. (**C**) *D. majalis*. (**D**) *D. maculata.* (**E**) ♀ *D. incarnata* × *D. fuchsii* ♂. (**F**–**G**) ♀ *D. fuchsii* × *D. incarnata* ♂. (**H**) ♀ *D. incarnata* × *D. majalis* ♂.

At 24 HAP, stages before and during megasporogenesis were noted: ovule protrusions with an archesporial cell and meiocytes at prophase I stage predominated (Fig. 4A); sometimes meiosis I was noted (Fig. 4D). Meiocytes at prophase I stage and later megasporogenesis stages were observed between 48 HAP and 5 DAP (Fig. 4B–C, E–G). At 5 DAP, the desynchrony in ovule development was more pronounced: ovules with the dyad, triad or FM (Fig. 4F) and a female gametophyte (Fig. 4G–H) were observed in the same ovary. In *D. maculata,* the deletion of chromosomes/bivalent from the metaphase I plate was noted (Fig. 4D). Gametogenesis progressed with each passing day from pollination. Polygonum-type embryo sac formed, with an egg apparatus at micropylar pole, two polar nuclei in central part and antipodal cells at chalazal pole of the embryo sac. Undisturbed pollen tube growth was observed (Supplementary Fig. S2A–C) and 10 DAP most of the ovules reached maturity. On day 21, viable ovules contained globular embryos (Fig. 5A–D).

### Seed viability

**Fig. 6:**
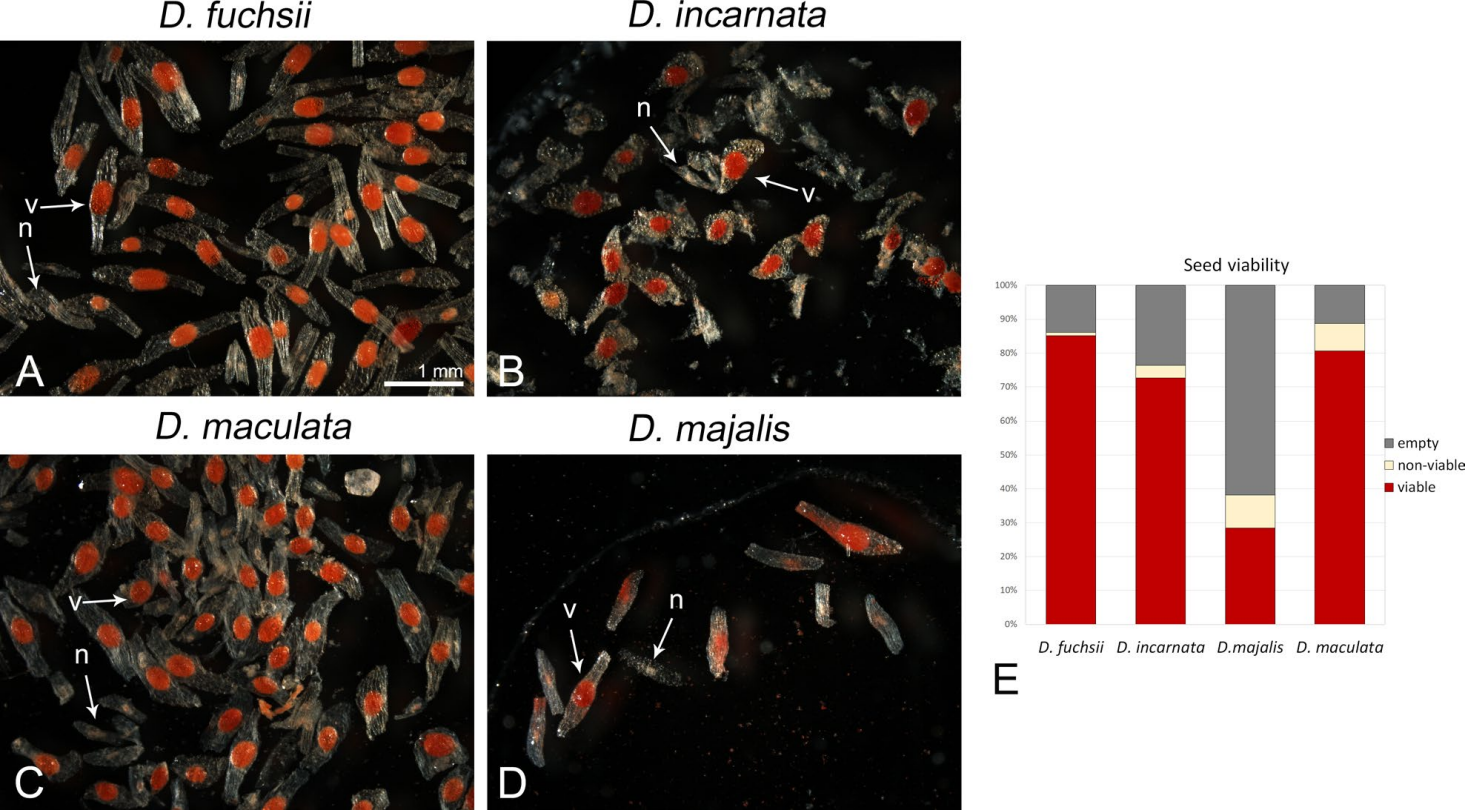
Seed viability (TTC) test for seeds collected from the allogamy experiment. (**A**–**D**) Embryos exhibiting reddish stain were classified as viable (v), while those that were uncoloured or exhibited yellowish tissue colouration were considered as non-viable (n). (**E**) Quantification of ovules noted among seed pool: viable, nonviable or empty (i.e., a lack of the embryo inside).

TTC test performed on seeds from open pollination and induced allogamy showed high viable seed production for diploids and *D. maculata* (*D. fuchsii*—85%, *D. incarnata*—73%, *D. maculata*—81%, respectively; Fig. 6A–E). More than half of the *D. majalis* seeds were empty (i.e., without an embryo, 62%); while 28% of the seeds contained viable embryos. Open pollination in the greenhouse generally failed due to lack of pollinators; however, when a flower was accidentally pollinated, seed production per inflorescence and seed viability were similar to those in allogamy.

### Interspecific crosses

Considering the differences in ploidy and evolutionary origin of the diploid species *D. incarnata* and *D. fuchsii* as parents of the tetraploid species *D. majalis*, we secondarily examined whether any abnormalities occurred after interspecific and interploid crosses. For all crosses, the stages of flower development between 24–48 HAP and up to day 5 were similar to those observed in allogamy; however, megasporogenesis proceeded faster in cross ♀ *D. fuchsii* × *D. incarnata* ♂ (Fig. 4I–L; Supplementary Table S1). Pollen germination and pollen tube growth inside the ovary were observed in flowers from all crossing experiments (Supplementary Fig. S2D–F). In 10 DAP, the first zygotes were recorded. On day 21 (21 DAP), the ovules either contained a few-cell embryo, or they were sunken/withered (Fig. 5E–H).

### Germination test of D. fuchsii/D. incarnata hybrid seeds

F_1_ crosses for ♀ *D. fuchsii* × *D. incarnata* ♂ and ♀ *D. incarnata* × *D. fuchsii* ♂ differed in the number of seeds per capsule and their morphology. At the first glance, the ♀ *D. fuchsii* × *D. incarnata* ♂ cross provided more seeds. The ♀ *D. incarnata* × *D. fuchsii* ♂ cross produced smaller bags and visually less seeds per capsule; the seeds and capsules were darker, and the bag was characterized by a thicker and impermeable shell, similar to *Platanthera* representatives (pers. comm. J. Znaniecka).

**Fig. 7:**
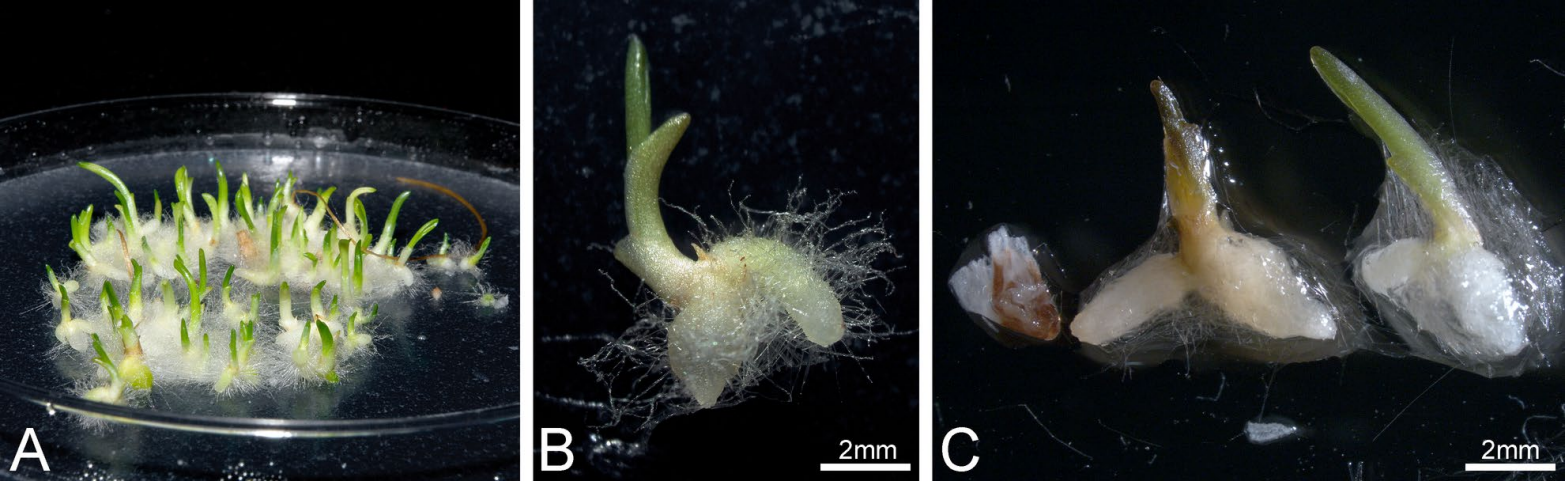
In vitro seedlings. (**A**, **B**) ♀ *D. fuchsii* × *D. incarnata* ♂ hybrid seedlings. (**C**) Three types of structures observed in vitro after germination of ♀ *D. incarnata* × *D. fuchsii* ♂ hybrid seeds: viable seedling (on the right), nonviable seedling (middle) and non-germinating seed (on the left).

Seeds from both crosses germinated well in vitro (Fig. 7A–C), with an efficiency of 100% (♀ *D. fuchsii* ×) and 70% (♀ *D. incarnata* ×). Prolonged soaking in Ca(OCl)_2_ had no effect on germination.

### Genetic differences of intercross hybrids

**Fig. 8:**
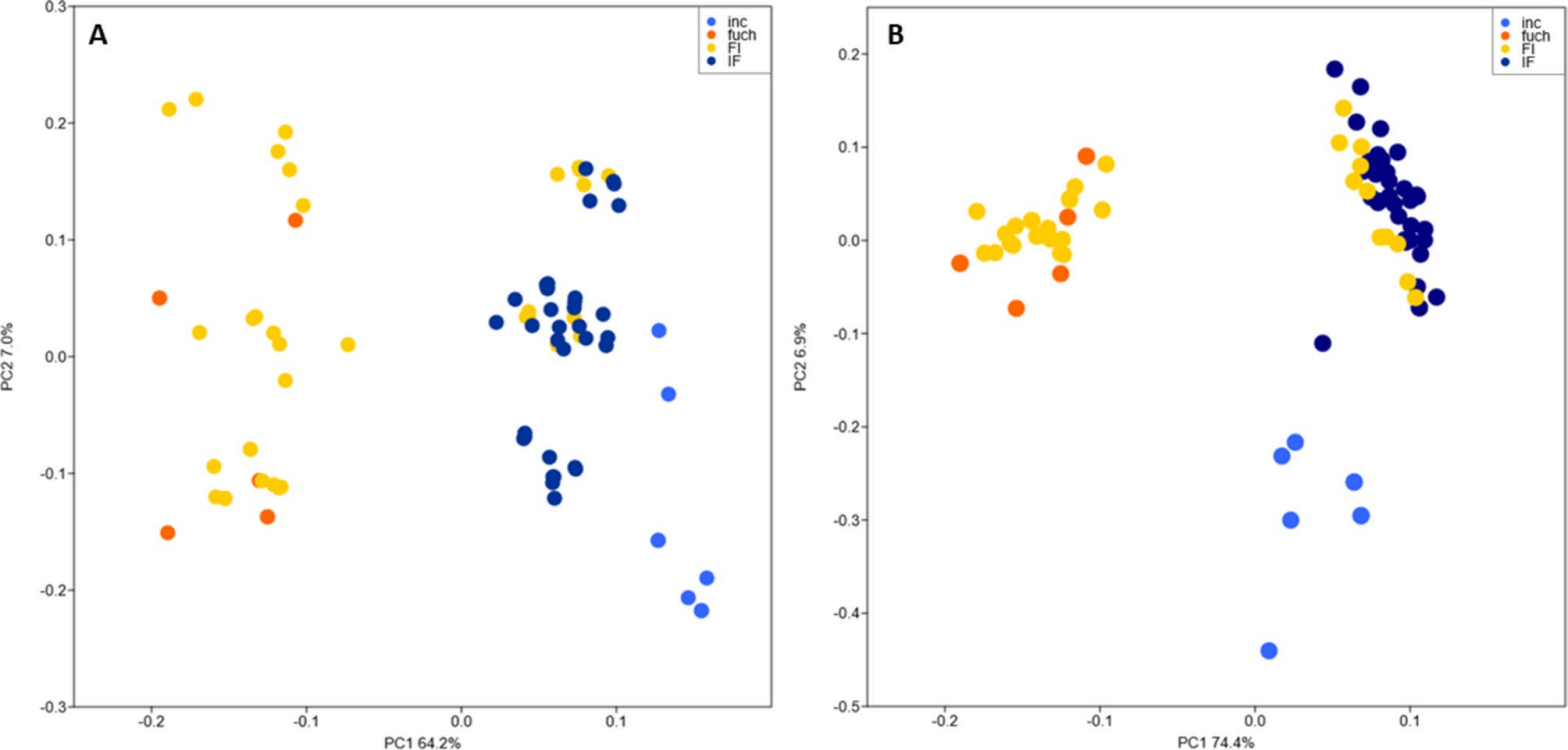
Principal coordinate analysis (PCoA) of microsatellite data from the parent species *Dactylorhiza* and their F_1_ hybrids. The ordination was based on Gower (**A**) and Bruvo (**B**) distance matrices between all individuals. Individuals are colored according to their parentage or one of two hybrid groups.

**Fig. 9:**
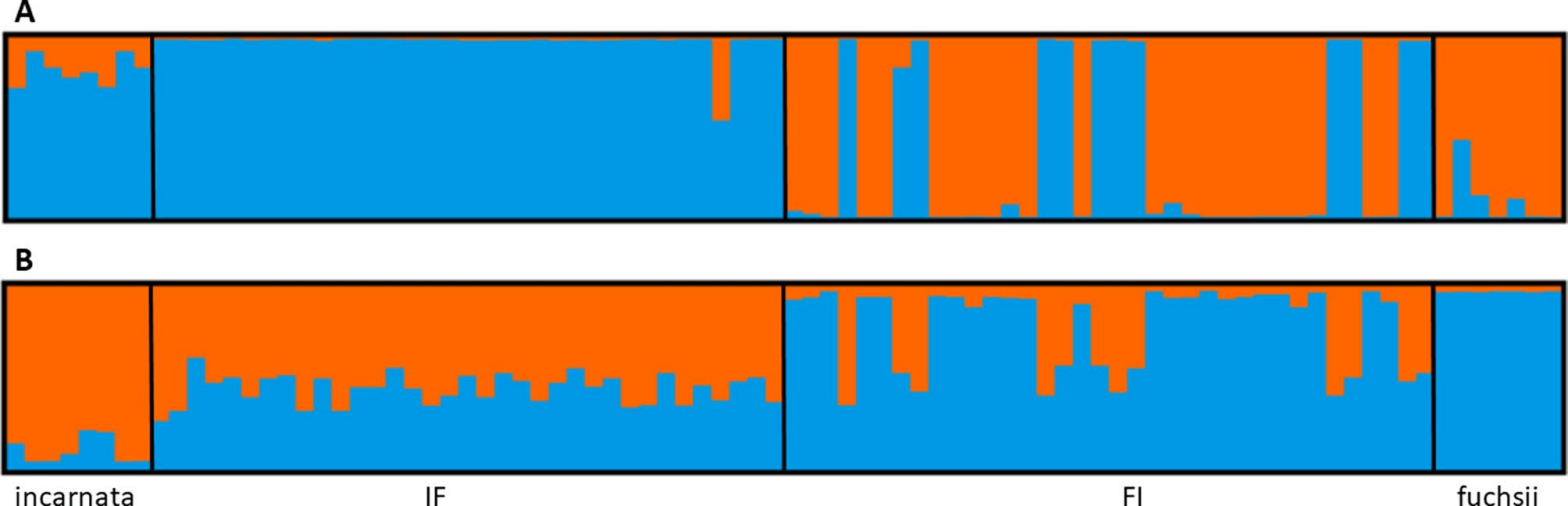
Bayesian admixture analysis using the parent species structure of *Dactylorhiza* and their F_1_ hybrids. Individuals were grouped into optimal (*K* = 2) log-likelihood partitions (**A**) and then analyzed using known origin as learning samples (**B**). Each bar represents a different individual, and the length of each segment is proportional to the estimated membership of each group.

Based on individual genetic distances, the parental species were clearly separated in the PCO plots (Fig. 8). Individuals classified as hybrids showed similarity to one of the parents. FI hybrids originated from crosses between♀ *D. fuchsii* × *D. incarnata* ♂ and IF hybrids from ♀ *D. incarnata* × *D. fuchsii* ♂, respectively. Some FI hybrids located on the left side of the plot, demonstrated resemblance to *D. fuchsii*, which functioned as the ovule parent during cross-pollination. In contrast, IF hybrids exhibited similarity associated with *D. incarnata*, which was the ovule parent here, but they were clearly genetically distinct from either parent, and were located on the right side of the plot. Eleven presumed FI hybrids did not show any similarity to the parental species *D. fuchsii*, but were genetically more similar to IF hybrids and the parental species *D. incarnata*. Clustering results for all individuals indicated a clear mode for *K*. The most likely number of clusters was determined to be *K* = 2 (Fig. 9). The first incarnata group was quite coherent, with a slight admixture, while the second group, which included the putative IF hybrids after crosses between *D. incarnata* ♀ × *D. fuchsii* ♂, had an almost homogeneous genetic background and was similar to *D. incarnata*. The third group was characterized by a high degree of admixture and FI hybrids after crosses between *D. fuchsii* ♀ × *D. incarnata* ♂ and showed genetic similarities to the fourth group of *D. fuchsii*. In the second clustering analysis, which employed parental species of known ancestry as a learning sample, genetically consistent groups of parental species were observed (Fig. 9B). However, the second group of IF hybrids indicated a higher degree of admixture than in the first analysis and showed an intermediate origin between the gene pools of the two parental species. In turn, the group with FI hybrids was more coherent, but still more similar to *D. fuchsii* and with a slight admixture from the second parent, *D. incarnata*.

Obtained plastid haplotypes confirm the maternal inheritance of cpDNA in the case of F_1_ hybrids (*D. incarnata* for IF hybrids and *D. fuchsii* for FI hybrids). Further markers revealed the presence of six different haplotypes in the total material (annotated HPL1–6), two of which were for *D. incarnata* (HPL5 and HPL6) and four for *D. fuchsii* (HPL1–4) (Table S1 in Supplementary Information). In the context of putative F_1_ hybrids, HPL6 was dominant and the only one haplotype for IF hybrids derived from the cross *D. incarnata* ♀ × *D. fuchsii* ♂. In turn, HPL3 was the predominant haplotype for FI hybrids derived from the cross *D. fuchsii* ♀ × *D. incarnata* ♂.

## Discussion

*Dactylorhiza incarnata/maculata* complex is characterized by frequent interspecific hybridization between diploid parental species, leading to the formation of tetraploid derivatives with distinct genome compositions^69,70^. This group serves as an excellent model system for investigating genomic and chromosomal changes following hybridization and polyploidization, offering valuable insights into how such rearrangements contribute to speciation and adaptation in polyploid taxa. The analysed *Dactylorhiza* diploids exhibited patterns of rDNA hybridization signals that are often observed among angiosperms, one pair of 5S rDNA loci and either one or two pairs of 35S rDNA loci, both in subterminal position^72^. The odd number of 35S rDNA loci observed in *D. fuchsii* and allotetraploid *D. majalis* might have resulted from intraspecific polymorphisms of the 35S rDNA loci, a phenomenon frequently reported in many plant species^73–75^. It is likely that one of the homologous chromosomes carried a 35S rDNA locus with a low number of repeat units that fell below the FISH sensitivity, resulting in hemizygosity of the detectable rDNA signal. Due to their conserved and tandemly repeated nature, 35S rDNA loci represent a significant source of genome instability. These loci are prone to unequal recombination and to recombination between nonhomologous chromosomes, especially when located in highly recombinogenic subtelomeric regions^76–78^. In the genome of the allotetraploid *D. majalis*, which originated through hybridization between *D. incarnata* and *D. fuchsii* followed by whole-genome duplication, the number of both 35S and 5S rDNA loci appears to be additive compared to the parental species, consistent with patterns reported for several relatively young allopolyploids^79,80^. In the karyotype of *D. maculata*, an autotetraploid species that originated from the diploid ancestor *D. fuchsii*, the number of 35S rDNA loci also appears additive. However, the number of 5S rDNA loci, one pair, is lower than expected. The number of rDNA loci frequently undergoes reorganisation in polyploid taxa. In most cases, a reduction in the number of 35S rDNA loci has been reported^81,82^. The re-patterning of 5S rDNA loci in polyploids has been documented; however, such reports are significantly less frequent than those involving 35S loci, and the vast majority of available data concern allopolyploid species^79,83^. Studies on rDNA evolution in autopolyploids, particularly natural ones, are relatively rare^84,85^. In autopolyploids, duplicated 5S rDNA loci located on homologous chromosomes and sharing identical non-transcribed spacer (NTS) sequences may undergo more efficient homogenisation. This process can promote unequal crossing over, potentially leading to the loss of some 5S rDNA loci. Available reports on autotetraploids suggest that the elimination of repetitive sequences and chromosomal rearrangements may contribute to the re-patterning of 5S rDNA loci^78,84,85^.

**Fig. 10:**
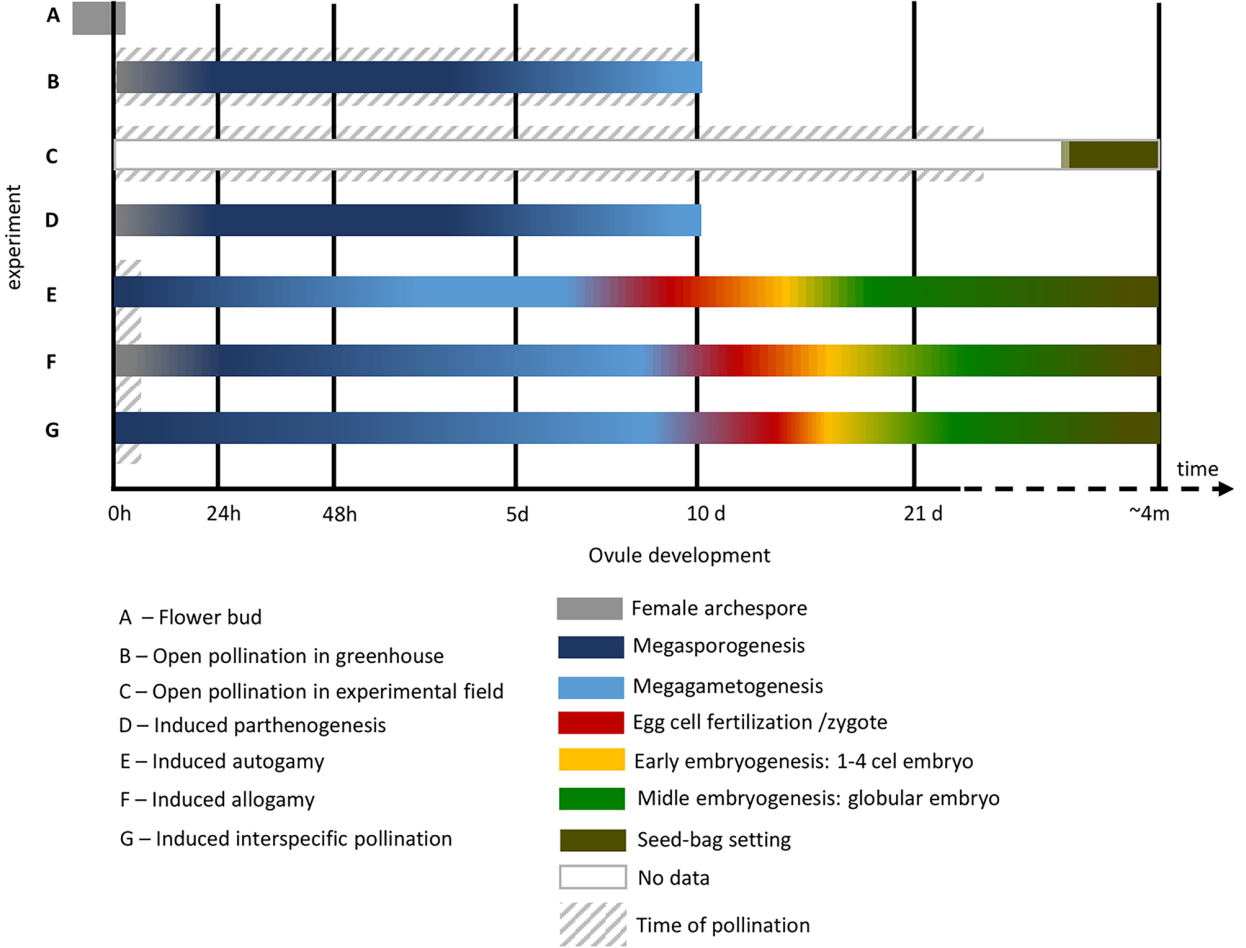
Ovules development in the *Dactylorhiza incarnata/maculata* complex under experimental conditions. Before anthesis, the ovules contained an archesporial cell (**A**). Open pollination in the greenhouse failed due to a lack of pollinators (**B**); however, seed-bags were formed when the plants were grown in an open experimental plot (**C**). Emasculated flowers that were isolated from pollination, degenerated by day 10, with ovules at the early stages of embryo sac development (**D**). All types of crossing (**E**–**G**) accelerated gametogenesis, and embryos and seeds formed successfully.

Reproductive barriers are strongly associated with the embryological processes occurring in developing flower buds and flowers. In this study, we proved embryologically that there are no prezygotic barriers preventing the formation of a hybrid embryo. However, observations of ovule development and genetic studies indicate that hybrid fitness can be modulated by postzygotic selection. Induced pollination accelerated the development of ovules and gametophytes regardless of the type or direction of crossing and led to the formation of embryos and seeds (Fig. 10D–E). Therefore, we found no evidence of prezygotic barriers in the *Dactylorhiza incarnata/maculata* complex. However, sporadic disturbances in meiosis in ovule plants, especially in *D. incarnata*, may be a source of unbalanced gametes and affect the fitness of offspring and their ploidy.

Our embryological studies confirmed previous field reports [e.g.^68^] indicating that visits by natural pollinators are necessary for successful open pollination in *Dactylorhiza* (Fig. 10B–C). The absence of pollinators in the greenhouse or a limited number of pollinators in the experimental plot hinders or prevents pollination. We observed a similar failure in the parthenogenesis experiment for *D. incarnata* (Fig. 10D). Interestingly, the ovules developed to some extent despite the lack of pollination, confirming the idea of its independence to some extent, at least regarding megasporogenesis. The similar observation was reported by Krawczyk et al.^49^ in the case of unpollinated flowers of *Epipogium aphyllum*, but there the embryo sac developed further, and parthenogenesis began. The low success in seed production for *D. majalis* after open pollination and allogamy may be due to its higher sensitivity to experimental conditions and/or its hybrid origin^44,86^. Additionally, reproductive success within *Dactylorhiza* (i.e., number of seed capsules, seed frequency and seed quality) varies by year/season [^87,88^pers. comm. J. Znaniecka].

We obtained viable hybrid seeds in interspecific crosses carried out in both directions, *D. incarnata* × *D. fuchsii* (which were able to germinate and develop to the protocorms after introduction into in vitro cultivation). This finding indicates that both diploid parent species have the potential to function as maternal or paternal plants. Similar observation was reported by Pinheiro et al.^89^, who revealed that hybridization can occur in both directions between *Epidendrum fulgens* × *E. puniceoluteum*. Furthermore, De hert et al.^21,36^ indicated that reproductive isolation appears to be asymmetric, depending on which *Dactylorhiza* species is the ovule or pollen parent, a phenomenon which has also been reported in other plant species^3,38,39^. Asymmetric hybridization is common in nature^37,90^ and has been documented in other orchid taxa, such as e.g., *Orchis*^38^ and in a pair of *Epidendrum* species (*E. madsenii* and *E. rhopalostele*)^91^. This finding stands in contrast to the prevailing understanding of the allopolyploid origin of many *Dactylorhiza* species, wherein *D. incarnata* is considered the predominant pollen parent rather than the ovule parent^31^. Conversely, crosses in both directions between the two parental species *D. incarnata* × *D. fuchsii* are less prevalent in nature than hybrids between the parental species and their tetraploid taxa^22^, which may suggest stronger reproductive isolation between the two diploid species than between the diploid and tetraploid species. However, we should bear in mind that the frequency of hybrids in nature may also be influenced by range, plant ecology, and phenology of the parental species^92,93^. *D. incarnata* and allotetraploid *D. majalis* more often share the same habitats and have a closer flowering time than either of them in relation to *D. fuchsii* (e.g.,^88,94^).

The absence of strong pre- and postmating barriers, as well as the ease with which seeds were produced after manual crossing, strongly suggest a hybridization mechanism in the studied complex. De hert et al.^21^ indicated that interspecific crosses yielded better results than intraspecific crosses. This outcome contrasts with numerous other studies which showed that interspecific pollination generated fewer or less viable seeds than intraspecific controls [e.g.,^8,17^] or that F_1_ hybrids exhibited reduced germination capacity^8^. In the present study, an embryological and genetic approach was utilized to demonstrate that prezygotic barriers in *Dactylorhiza* species are relatively weak in comparison to slightly stronger postzygotic barriers. These results are consistent with previous observations [e.g.,^21,24,27,31,36,95^]. The exclusion of apomictic development in the *Dactylorhiza incarnata/maculata* complex was confirmed by the absence of parthenogenetic embryos (Fig. 10D). Furthermore, our observations indicate that pollination per se is not required for megasporogenesis and early stages of gametogenesis, but that the embryo sacs degrade before reaching maturity. This finding may indicate that tactile manipulation (in this case emasculation) can trigger maternal epigenetic and hormonal signals that mimic pollination^96–98^. Therefore, it is unlikely that apomixis is the presumed mechanism for the emergence of a new phenotype in the study complex.

The disturbances in chromosome segregation during meiosis in *D. incarnata* and *D. maculata* suggest that changes in the number of chromosomes in gametes may occur even before fertilization and generate F_1_ cytotypes with a certain degree of sterility. Such abnormalities may lead to aneuploid chromosome numbers in female gametes, which may provide a plausible explanation for seed sterility^44^. Chromosomal rearrangements have been shown to disrupt normal chromosome pairing, resulting in a deficiency of recombination and abnormal segregation during meiosis in hybrid progeny. This, in turn, leads to the production of sterile gametes, thereby reducing the fertility of F_1_ hybrids^99,100^. It has been established that many genes are expressed in male gametes of plants, and pollen containing chromosomal abnormalities is more likely to be aborted than female gametes, which are more tolerant to chromosome rearrangements^101,102^. In addition, several meiotic abnormalities have also been observed in *Epidendrum* plant hybrids. These include unpaired chromosomes at prophase I/metaphase I, early separation at metaphase I and anaphase I, and a plate metaphase split into two parts at metaphase I. These findings suggest a role for chromosomal rearrangements in hybrid sterility^103,104^.

The majority of studies on the subject of orchid hybridization have demonstrated that hybrids typically occupy an intermediate morphological and genetic position relative to their parental species, thereby suggesting that they combine characteristics of both parents^33,34,36,93^. These results are consistent with earlier reports on orchids, in which hybrids also exhibited intermediate traits [e.g.,^13,14,105,106^]. On the other hand, there is increasing evidence that in orchids, the maternal parent contributes considerably to the offspring^107^. In our study, individuals classified as hybrids showed resemblance to one of their parents in the STRUCTURE analysis. Specifically, IF hybrids exhibited similarities to *D. incarnata*, which in this case was the ovule parent. All IF hybrids can be interpreted as F_1_ hybrids. In particular, this is easy to determine because each individual contains approximately equal proportions of the two clusters, that correspond to the respective parental species. In contrast, FI hybrids showed similarities mainly to *D. fuchsii*, which functioned as the ovule parent during cross-pollination. However, some FI specimens show a genetic admixture of both clusters and overlap with IF hybrids in the PCoA analysis, indicating that they are undoubtedly F_1_ hybrids. The remaining FI hybrids are classified within cluster corresponding to *D. fuchsii*, and also overlap with this species in the PCoA analysis. Perhaps, these individuals are the result of parthenogenesis in *D. fuchsii* (what was not tested), induced by pollination with heterospecific pollen. On the other hand, endopolyploidization causes the alleles of one parent to multiply in the genome, while the other parent is suppressed. It is a cellular process in which genetic material (chromosomes) is duplicated without the simultaneous division of the cell nucleus and cytoplasm. This results in the formation of polyploid cells, i.e. cells that have more than two sets of chromosomes^108^. This result remains difficult to interpret, and it cannot be ruled out that the observed pattern is characteristic of this Pomeranian parental population of *D. fuchsii* selected for the study. Nevertheless, more such populations would be necessary to confirm the obtained pattern. Moreover, insight into detailed transcriptomic studies of *Dactylorhiza* provide a crucial source of information for understanding post-hybridization evolution and whole-genome duplication, phenomena that are particularly common in this genus. Extensive molecular divergence between the two parental species of *Dactylorhiza* suggests a considerable genomic and transcriptomic shock in their hybrids (i.e., “genomic shock”^109^) and may help to understand the difficulties of coexistence of these two genomes at the homoploid level^69,110^. These differences cause uneven expression patterns between parental subgenomes, ultimately leading to the preferential degradation of one of the homoeologs^71,110^.

Previous crosses had already suggested that *D. fuchsii* may control seed fertility expressed in the natural hybrid *D. majalis* through an unknown maternal mechanism. Such crosses produced slightly more viable seeds when *D. fuchsii* served as the pollen recipient and *D. incarnata* as the pollen donor, compared to the reverse crosses^21,44^. On the other hand, it has also been speculated that the morphology of the allotetraploid depends on which of the parental species acts as the female parent and provides the chloroplast genome. In consequence, diverse combinations of male and female parents would result in offspring with different appearances. Indeed, synthetic allopolyploids in *Brassica* have been shown to shift towards the female parent in successive generations^111^. This is presumably a strategy to avoid problems with chloroplast and mitochondrial genome compatibility^112^. In the case of *Dactylorhiza* allotetraploids, they are generally more similar to *D. incarnata* s.l. than to *D. fuchsii/maculata*, which may indicate that the former often served as the female parent^24^. This contrast with the allopolyploid origin of many *Dactylorhiza* species^31,45^. However, the greater similarity to *D. incarnata* can also be explained by the higher level of homozygosity and lower genetic variability in *D. incarnata* s.l. than in *D. fuchsii/maculata* lineages^30^. The genetic structure of both species has been shaped by different histories, because unlike *D. fuchsii*, *D. incarnata* has experienced a dramatic decline in population size, which has resulted in a homogenization of its genetic variation in neutral markers^24,31,57^, despite its current wide distribution and considerable morphological heterogeneity. The directional influence of the female parent on the morphology of hybrid has been described repeatedly, along with problems of compatibility between nuclear and organelle genomes^113,114^, where a shift towards the maternal parent (‘maternal bias’) influences, among other things, the expression of allopolyploid genes^115^.

Furthermore, Orchidaceae do not possess endosperm in their seeds, which may facilitate crossing in both directions, as suggested by Moraes et al.^103^ in their study on the formation *Epidendrum fulgens* × *E. puniceoluteum* hybrids. In other words, the ability of orchid embryos to develop without an endosperm removes one of the potential barriers to hybridization failure^116^. Moreover, the presence of pollen grains as a cohesive mass of dozens of tetrads, i.e., pollinium, may have a direct impact on the reproductive success of hybrids by minimizing gamete loss^117,118^. Since pollinium is deposited on the stigma as an entire unit, this may reduce pollen loss. Highly abnormal hybrid meiosis, which should produce only a few viable female gametes, can be compensated for by the massive deposition of pollen, which should pollinate all viable female hybrid gametes^103^.

## Conclusions

Our research has shown that reproductive isolation in representatives of the *Dactylorhiza incarnata/maculata* complex is regulated primarily by postzygotic mechanisms rather than prezygotic ones. Embryological and cytogenetic analyses revealed irregular meiosis, reduced fertility and variable seed viability in diploid and tetraploid taxa. Cross-hybridization between *D. incarnata* and *D. fuchsii* confirmed that hybridization can occur bidirectionally, albeit with asymmetrical success rates. Fluorescent in situ hybridization revealed species-specific rDNA rearrangements consistent with both auto- and allopolyploid origins within the group. These results challenge the long-held assumption that *D. incarnata* consistently acts as the pollen parent in the formation of allotetraploids, revealing instead more complex and dynamic patterns of hybridization. In summary, the results indicate weak prezygotic barriers, strong postzygotic constraints, and cytogenetic instability as key factors shaping species boundaries, while repeated hybridization and polyploidization appear to be the main evolutionary forces driving diversification and genetic richness in orchids of the genus *Dactylorhiza*.

## Supplementary Information

Supplementary Information 1.

Supplementary Information 2.

## Data Availability

All additional images, raw data (as Supplementary spreadsheets), tables supporting the presented results are included as supplementary files. Voucher specimens have been also deposited in the UGDA Herbarium (https://herbarium.ug.edu.pl/en/herbarium-universitatis-gedanensis-ugda/).
