# Supplementary material for: Hybridization and embryological patterns underpinning reproductive barriers in Dactylorhiza (Orchidaceae)
Source: Sci Rep. 2025 Oct 22;15:36824. doi: 10.1038/s41598-025-24871-2 (PMC12546816; doi:10.1038/s41598-025-24871-2)
Supplement: Supplementary file 1 — Supplementary Information 1. [file 41598_2025_24871_MOESM1_ESM.pdf]

Reproductive barriers and hybridization in the *Dactylorhiza incarnata/maculata* complex: an embryological and genetic perspective

Aleksandra M. Naczk[1], Małgorzata Kapusta[2], Bożena Kolano[3], Joanna Znaniecka[4], Katarzyna Meyza[5], Marcin Górniak[6], Joanna Rojek[7*]

Supplementary files:

Figure S1.

Figure S2.

Table S1.

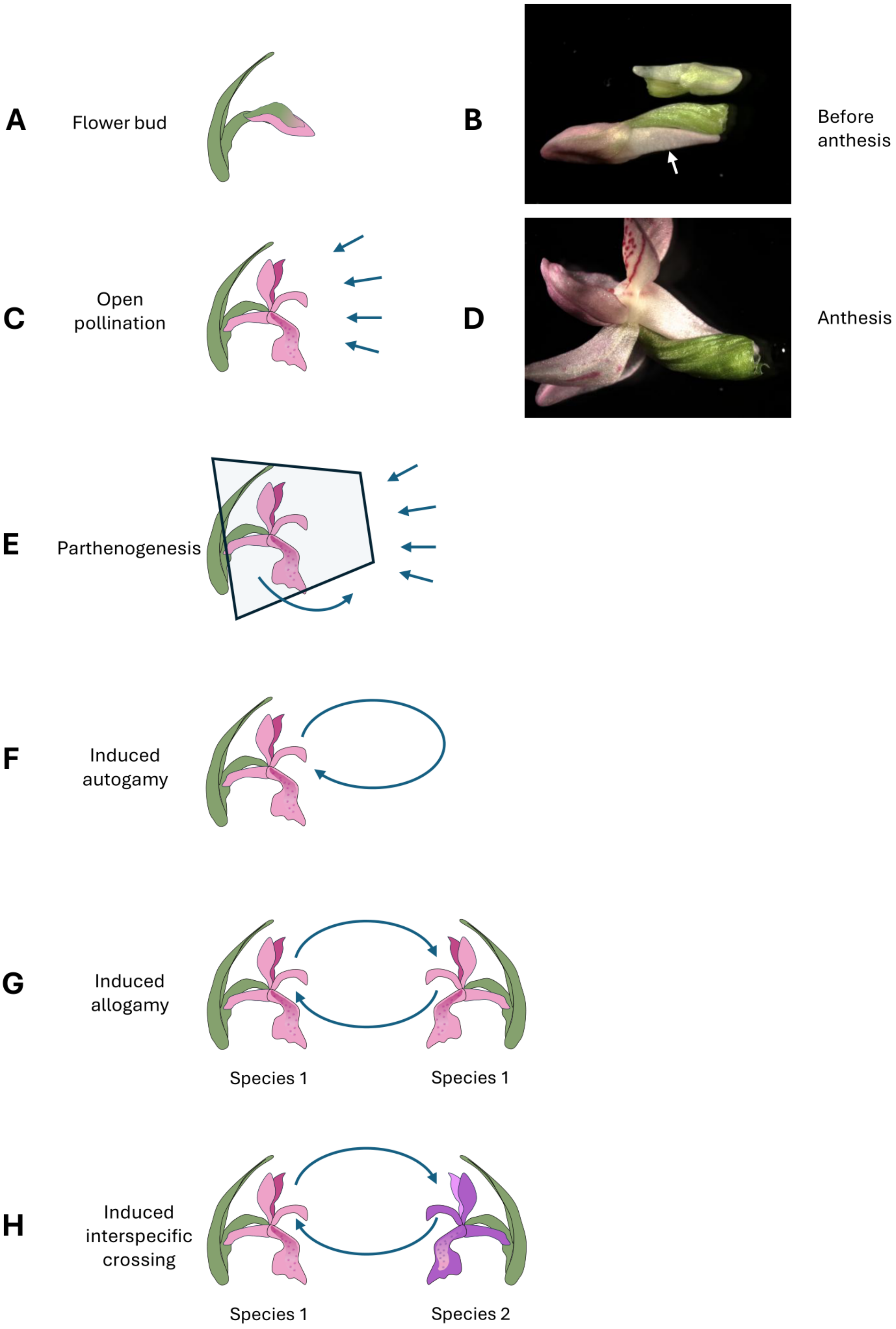

Figure S1. Experiment overview. (A–B) Flower bud used for embryological studies before anthesis experiment (white arrow in B). (C) Pollination could have occurred accidentally (phytotron free of potential pollinators) or by pollinating insects (experimental plot variant). (D) The flower stage for C–H experiments. (E) Emasculated flower (arrow pointing outwards) was covered with a mesh bag to prevent incidental pollination. (F) Emasculated flower was then hand-pollinated with its own pollen. (G) Emasculated flower was then hand-pollinated with foreign pollen of the same species. (H) Emasculated flowers were then hand-pollinated with foreign pollen from another species. Blue arrowheads indicate the pollen vector.

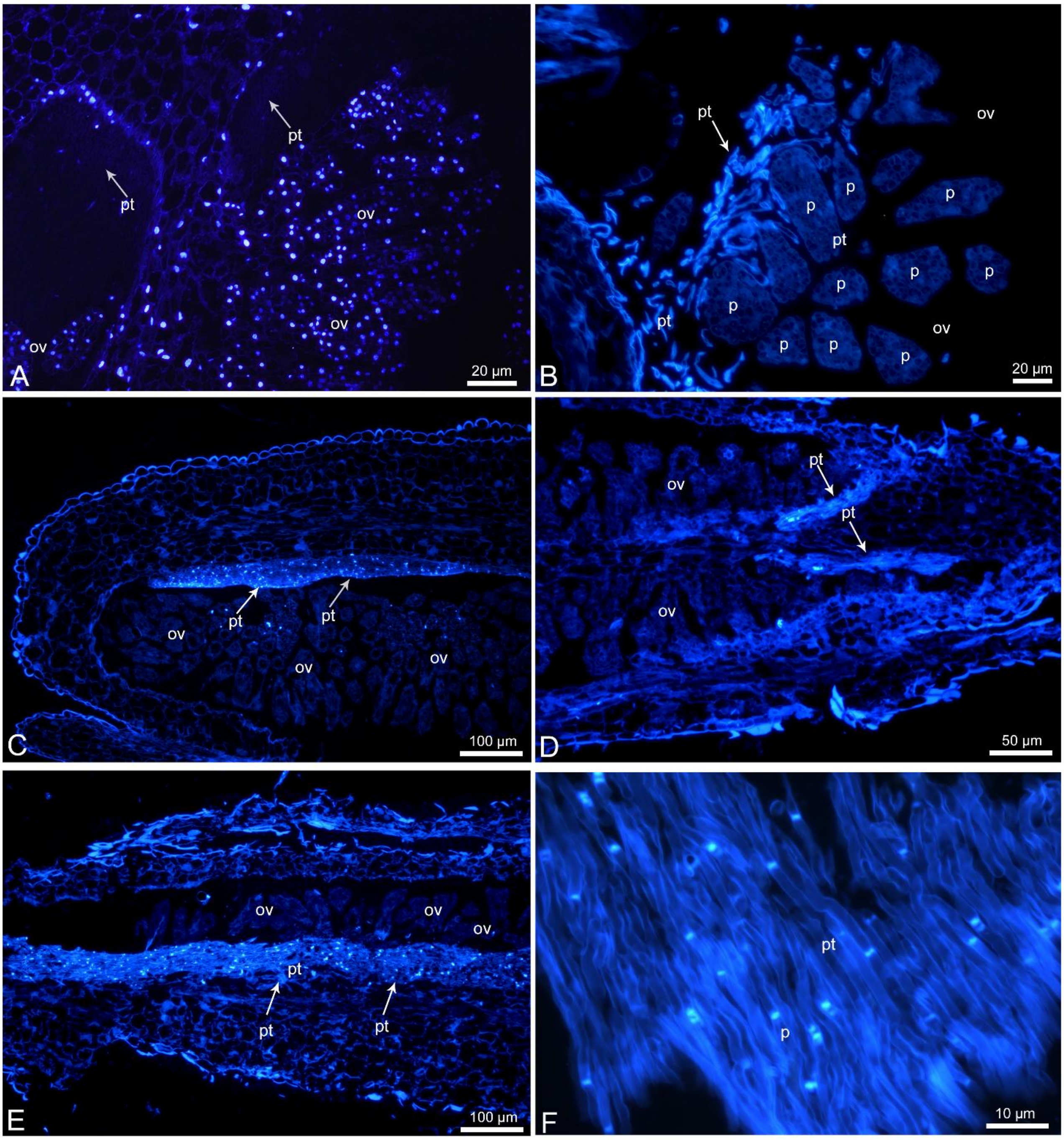

Figure S2. Representative photographs of pollen tubes growing into the ovaries. (A) *D. maculata* pollen tubes (pt) growing into the ovary and visible ovules (ov) in the ovary at day 10 after hand pollination (allogamy). (B) *D. majalis* pollen tubes (pt) growing out of pollinia (p) on day 5 after hand pollination (allogamy). (C) *D. fuchsii* ♂ pollen tubes (pt) growing into the ovary and visible ovules (ov) in the ovary at day 7 after hand pollination (allogamy). (D) Pollen tubes of *D. incarnata* ♂ (pt) growing into the ovary and visible ovules (ov) in the ovary of *D. fuchsii* ♀ at day 10 after hand pollination. (E) Pollen tubes of *D. majalis* ♂ (pt) growing into the ovary and visible ovules (ov) in the ovary of *D. incarnata*♀ at day 10 after hand pollination. (F) Pollen tubes of *D. incarnata* ♂ growing into the ovary of *D. majalis* ♀ at day 7 after hand pollination.

Table S1. Developmental survey of flowers after interspecific crossing within the *Dactylorhiza incarnata/maculata* complex. h-hours after cross-pollination; d-days after cross-pollination.

Cross direction	Developmental stage			
	Megasporogenesis	Megagametogenesis	Fertilization/zygote	Embryogenesis
D. fuchsii ♀× *D. incarnata* ♂	24 – 48 h	48 h – 10 d	10 – 14 d	14 – 21 d
D. fuchsii ♀ × *D. majalis* ♂	24 h – 7 d	7 – 14 d	14 – 21 d	21 d
D. incarnata ♀ × *D. fuchsii* ♂	24 h – 5 d	48 h – 10 d	14 d	14 – 21 d
D. incarnata ♀ × *D. majalis* ♂	24 h – 5 d	48 h – 14 d	14 d	21 d
D. majalis ♀ × *D. incarnata* ♂	24 h – 5 d	5 – 7 d	10 d	14 – 21 d
D. majalis ♀ × *D. fuchsii* ♂	24 h – 5 d	5 – 10 d	10 – 14 d	14 – 21 d
